# Microbial Cell Factories in the Bioeconomy Era: From Discovery to Creation

**DOI:** 10.34133/bdr.0052

**Published:** 2024-10-21

**Authors:** Xiongying Yan, Qiaoning He, Binan Geng, Shihui Yang

**Affiliations:** State Key Laboratory of Biocatalysis and Enzyme Engineering, and School of Life Sciences, Hubei University, Wuhan 430062, China.

## Abstract

Microbial cell factories (MCFs) are extensively used to produce a wide array of bioproducts, such as bioenergy, biochemical, food, nutrients, and pharmaceuticals, and have been regarded as the “chips” of biomanufacturing that will fuel the emerging bioeconomy era. Biotechnology advances have led to the screening, investigation, and engineering of an increasing number of microorganisms as diverse MCFs, which are the workhorses of biomanufacturing and help develop the bioeconomy. This review briefly summarizes the progress and strategies in the development of robust and efficient MCFs for sustainable and economic biomanufacturing. First, a comprehensive understanding of microbial chassis cells, including accurate genome sequences and corresponding annotations; metabolic and regulatory networks governing substances, energy, physiology, and information; and their similarity and uniqueness compared with those of other microorganisms, is needed. Moreover, the development and application of effective and efficient tools is crucial for engineering both model and nonmodel microbial chassis cells into efficient MCFs, including the identification and characterization of biological parts, as well as the design, synthesis, assembly, editing, and regulation of genes, circuits, and pathways. This review also highlights the necessity of integrating automation and artificial intelligence (AI) with biotechnology to facilitate the development of future customized artificial synthetic MCFs to expedite the industrialization process of biomanufacturing and the bioeconomy.

## Introduction

Biomanufacturing refers to the use of a biological system composed of enzymes, microorganisms, or more advanced biological cells to manufacture diverse products with economic value, such as biofuels, biochemicals, nutrients, natural products, or other value-added products from renewable feedstocks, such as lignocellulosic biomass, via biotechnology [1]. MCFs are the “chips” of biomanufacturing. The design and construction of efficient and robust MCFs have the potential to meet the needs of environmental protection, carbon neutralization, and global economic development toward the coming sustainable bioeconomy. The concept of metabolic engineering was first officially suggested in 1991 with the development of molecular biology and genetic engineering tools to design metabolic pathways in organisms [2]. The development of next-generation sequencing (NGS) and spectrometry technology has enabled a systems biology approach for the deep understanding of microbial metabolic networks and regulation, which has been applied to the construction of higher-level MCFs [3]. The emergence of synthetic biology in combination with the accumulation of systems biology datasets and the development of automation and big data analysis technology have taken MCFs into a design or synthetic era. The integration of artificial intelligence (AI), automation, and synthetic biology with systems biology has led to the thorough understanding and modification of many chassis cells to enable the rational design and modification of both model and nonmodel chasses into robust and efficient cell factories capable of producing natural and nonnatural products of interest [4,5].

Although bioproducts such as biofuels, bioplastics, and biomedicines have been produced industrially via microbial fermentation, challenges persist regarding production scalability and cost. Therefore, the use of low-cost biomass feedstock for platform biochemical production and the development of MCFs with improved production performance and robustness are crucial to address the industrialization problems. To increase the production capacity of MCFs to meet industrial requirements, it is essential to reprogram and optimize model and nonmodel chasses to develop robust and efficient cell factories for industrial applications.

The accumulation of omics datasets and advancements in cutting-edge technologies have made it possible to analyze and interpret the metabolic pathways, regulatory networks, and mechanisms of microbial chassis cells in detail. Multidimensional rational or semirational design of microbial chassis cells can be performed by combining the design-build-test-learn (DBTL) strategy with synthetic biology tools. This involves exploring and designing biological parts, circuits, and modules to construct metabolic and regulatory pathways and then optimizing them for rational design and industrial development of diverse bioproducts by integrating spatiotemporal regulation [6].

**Fig. 1.**
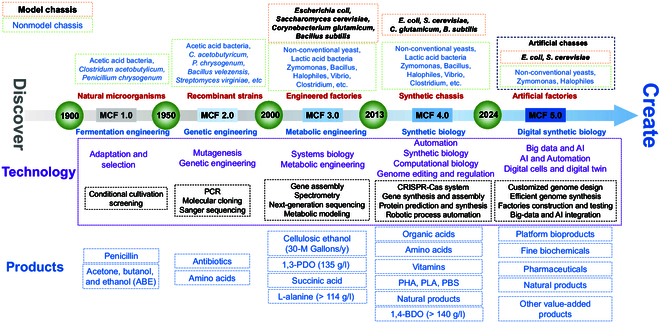
Development of MCFs from discovery to creation.

In this review, we highlight the importance of understanding microbial chassis cells and provide a timeline-based overview of the advancements and strategies employed in the development of MCFs. We divide the chronology of the MCFs into 5 distinct periods and provide a detailed overview of the characteristics of each period (Fig. 1). The discovery and development of natural cell factories, as well as the creation of future artificial cell factories, are discussed. In addition, we summarize the advancements and strategies in the construction of robust and efficient MCFs with balanced and optimized metabolism in terms of substance, energy, physiology, and information. We also highlight the importance and necessity of synthetic biology strategies and corresponding enabling technologies in the construction and optimization of MCFs. Moreover, this review highlights the necessity of integrating automation and information technology (IT) with biotechnology to facilitate the development of future customized artificial synthetic MCFs that will help accelerate the advancement of biomanufacturing and the bioeconomy.

## Development of Industrial Chassis Cells

The isolation and discovery of natural MCFs was followed by the generation of cell factories with superior performance through mutagenesis and genetic engineering. With advances in biotechnology, rational methods such as metabolic engineering have been applied to modify chassis cells to achieve more desirable characteristics. The emergence and development of synthetic biology have facilitated the establishment of technology platforms. It can be used not only for better understanding model and nonmodel microorganisms but also for guiding the rational design, synthesis, assembly, and construction of robust and efficient cell factories. Moreover, the accumulation of biological data and the continuous development of AI will guide the development of digital biology, which involves the application of digital methods to study and quantify cell factories from genes, circuits, and pathways to ecosystems [7]. Even in the development of future cell factories, information sharing between different species, such as some model and nonmodel chasses, can be achieved through digital synthetic biology, ultimately leading to the development of synthetic artificial chasses (Fig. 1). The representative products, including foods, fuels, chemicals, and medicines synthesized from different generations of MCF, are summarized in Table 1. However, few related reports about digital cells as a future development trend exist.

**Table 1.** Summary of representative products synthesized from different generations of MCFs

Class	Host	Target chemical	Strategy description	Titer (g/l)	Reference
MCF 1.0	*P. chrysogenum*	Penicillin	Strain selection and isolation	0.06	[14]
	*C. acetobutylicum*	Butanol	Strain selection and isolation	10–12	[157]
MCF 2.0	*P. chrysogenum*	Penicillin	X-ray mutagenesis	0.3	[14]
MCF 3.0	*E. coli*	l-Alanine	Heterologous alanine dehydrogenase integration, competitive pathway deletion	114	[35]
	*E. coli*	1,3-Propanediol	Pathway design and optimization, cofactor supply	130	[158]
	*S. cerevisiae*	Artemisinic acid	Metabolic regulation, optimization of key genes expression levels, competitive pathway deletion	0.1	[106]
	*C. glutamicum*	l-Valine	Cofactor balance, competitive pathway deletion	150	[159]
	*B. subtilis*	Riboflavin	Mutagenesis of *zwf* and *gnd*	15.7	[160]
	*C. acetobutylicum*	Butanol	Overexpression of *groESL*	17	[157]
MCF 4.0	*E. coli*	1,4-Butanodiol	Pathway design, energy supply, cell growth, redox balance	18	[39]
	*S. cerevisiae*	Cannabinoids	Enzyme mining, copy number optimization	/	[107]
	*Y. lipolytica*	Succinic acid	Reconfiguration of the reductive TCA, adaptive laboratory evolution	111.9	[66]
	*H. bluephagenesis*	Polyhydroxyalkanoates (PHA)	Plasmid copy number optimization, cell morphology regulation	80	[100]
	*Z. mobilis*	Polyhydroxybutyrate (PHB)	Copy number optimization, cofactor supply, self-flocculation, C/N ratio	74% DCW	[87]
	*B. licheniformis*	2-Phenylethanol	Central metabolic pathway and phenylpyruvate pathway reconstruction, competitive pathway deletion, glucose transport system modulation	6.24	[161]
	*Rhodotorula toruloides*	Fatty acid ethyl esters	Overexpression of wax ester synthase genes	9.97	[162]

## Isolation and Discovery of Natural Industrial Microorganisms

Five thousand years ago, the discovery of the sour liquid produced by the natural fermentation of fruits opened the way to the understanding and use of vinegar. Vinegar plays an important role in the culinary, health, beverage, and chemical industries as well as other fields. Its production uses specialized aerobic acetic acid bacteria (AAB) to express ethanol dehydrogenase and acetaldehyde dehydrogenase, which oxidize the alcohol produced from grain or fruit fermentation to acetic acid. *Acetobacter*, the first AAB discovered, has industrial importance in edible vinegar production in the food industry. With the ongoing advancements in biotechnology, an increasing variety of AAB have been isolated [8] and widely applied in the production of cellulose, pigments, indole acetic acid, and ascorbic acid [9–11].

In the 20th century, energy shortages led to the rapid development of biobutanol, which is an important chemical feedstock and biofuel. *Clostridium acetobutylicum* has been extensively studied as an important strain for classical natural butanol production [12]. *C. acetobutylicum* can effectively undergo continuous industrial fermentation for acetone, butanol, and ethanol production at a ratio of 6:3:1 by using renewable biomass resources such as starch and molasses [13]. However, the high cost limits the development of butanol from microbial cells; thus, breeding high-proportion butanol strains and developing new fermentation processes are pivotal challenges.

The discovery of penicillin opened a new path for the use of antibiotics to treat infectious diseases, which marked a new era of antibiotics and drugs for humanity. The use of *Penicillium chrysogenum* made industrial fermentation production of penicillin a reality. However, the low titer of penicillin (~5 mg/l) led to an unaffordable price due to limitations in upstream strain engineering, fermentation optimization, and downstream processing for production isolation and purification [14]. Since the discovery of penicillin, more than 20,000 biologically active antibiotic drugs have been discovered in microorganisms [15]. For example, *Cephalosporium acremonium* was identified as a cephalosporin producer and actinomycetes as cephamycin, clavam, and carbapenem producers and, along with unicellular bacteria, as monocyclic β-lactam producers.

In addition, an increasing number of natural producers of biochemicals have been isolated and discovered, such as *Anaerobiospirillum succiniciproducens* and *Mannheimia succiniciproducens* for succinic acid [16,17], *Rhodococcus opacus* and *Yarrowia lipolytica* for lipids, fatty acids, and their derivatives [18,19], *Corynebacterium glutamicum* for amino acids [20], *Zymomonas mobilis* and *Saccharomyces cerevisiae* for ethanol [21,22], *Lactobacillus* for lactic acid [23], and *Klebsiella pneumoniae* for 1,3-propanediol (1,3-PDO) [24]. These naturally acquired strains have become the first generation of industrial microbial cell factories (MCF 1.0). However, it cannot completely meet the needs of industrial applications in terms of product titer, rate, and yield. The development of microbial chassis cells with better characteristics is critical.

## Random Mutagenesis and Natural Selection

Owing to the limited information and genetic tools available for microorganisms selected from natural environments, random mutagenesis, which includes physical, chemical, and biological mutagenesis, was the primary method used to improve the features of these strains. The development of cell factories through mutagenesis and selection marked the second generation of industrial microbial cell factories (MCF 2.0).

### Physical and chemical mutagenesis

**Fig. 2.**
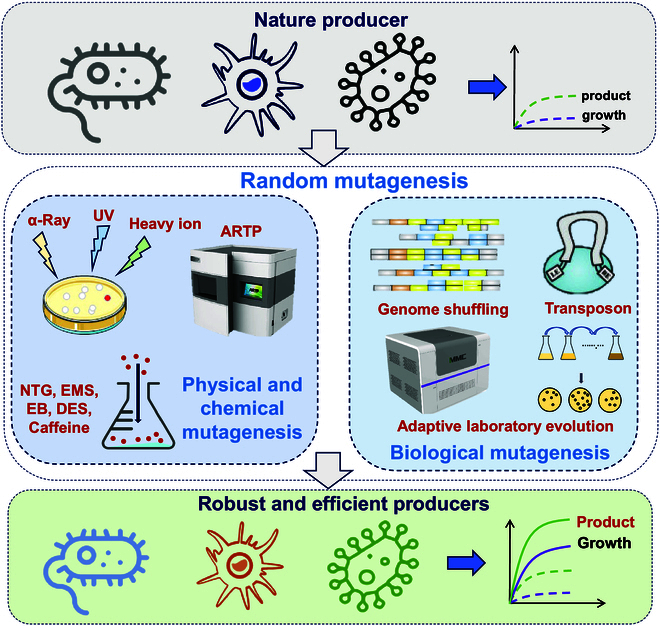
Strategies of random mutagenesis for natural MCF evolution

Physical mutagenesis mainly uses physical factors, including ultraviolet (UV) light, x-rays, α-rays, γ-rays, and heavy ion irradiation, to induce mutations (Fig. 2). Recently, a novel mutagenesis tool, atmospheric and room temperature plasma (ARTP), which is a powerful mutagenesis tool to improve the yield of biochemicals, was developed. The advent of ARTP has dramatically enhanced the quality and characteristics of products derived from bacteria and fungi that are difficult to modify, such as antibiotics, organic acids, amino acids, vitamins, and steroids [25].

The mutants obtained through mutagenesis are listed in Table 2. Multiple mutants were acquired through mutagenesis, with enhanced bacteriocin production ranging from 103.48% to 551% [25], and an l-serine producer with a titer of 34.78 g/l was obtained via ARTP mutagenesis [26]. Chemical mutagenesis primarily uses chemical substances such as alkylating agents, base analogs, frameshift mutagens, deamination agents, and hydroxylation agents to obtain mutations. Although chemical mutagenesis provides high mutation efficiency, its use of toxic mutagens poses risks to human health and the environment. Therefore, the use of physical or biological mutagens will likely be the main trend in the future.

**Table 2.** Examples of the application of physical and chemical mutagenesis to improve production

Strain	Method	Product	Improvement (-fold)	Ref.
*Actinobacillus succinogenes*	UV + NTG	Succinic acid	1.85	[163]
*Propionibacterium acidipropionici*	UV	Propionic acid	1.25	[164]
*Bacillus velezensis*	DES + NTG	Surfactin	3.99	[165]
*Streptomyces virginiae*	UV	Virginiamycin	11.6	[166]
*Cordyceps kyushuensis*	UV + HNO_2_	3β,7α,15α-Trihydroxy-5-androsten-17-one	9.6	[167]
*Actinosynnema pretiosum*	ARTP	Ansericin	1.93	[168]

### Biological mutagenesis

Biological mutagenesis has led to innovations in mutation breeding. This not only accelerates mutagenesis efficiency but also ensures safety. This approach mainly includes techniques involving bacteriophages, plasmids, DNA transposon mutagenesis, and protoplast fusion, such as DNA and genome shuffling. Genome shuffling integrates traditional strain mutagenesis breeding methods with protoplast techniques, enabling the convergence of superior phenotypes from multiple parents into a single strain through multiple rounds of protoplast fusion. This approach accelerates the forward mutation process greatly in microbial cells. As a result, it has been widely applied to create strains for bioproducts with complicated pathways, such as the biofuels butanol and ethanol [27]; antibiotics such as doxorubicin [28], avilamycin [29], and nosipeptide [30]; and natural food preservatives such as ε-poly-l-lysine [31]. In addition, genome shuffling has also been used to improve other microbial traits, such as byproduct reduction. For example, genome shuffling was successfully used to reduce the glycerol byproduct in a mutant of industrial *S. cerevisiae* while increasing the tolerance to ethanol and inhibitory byproducts such as furfural and acetic acid in lignocellulosic hydrolysates [32,33].

Random mutagenesis plays an important role in the breeding of efficient MCFs. However, the mutations are highly random across the entire genome, leading to difficulty in obtaining the desired traits rapidly. Therefore, rational design and engineering of microbial chassis cells into efficient cell factories has become increasingly important.

## Metabolic Engineering of MCFs

The success of the Human Genome Project and technological advances have led to a deeper understanding of microbial chassis cells at the genomic level. The accumulation of omics datasets and improvements in genetic tools have made genetic modifications in model microorganisms efficient for the production of diverse bioproducts, such as organic alcohols and acids, amino acids, organic amines, vitamins, natural products, and polyhydroxyalkanoates (PHAs). Research directions therefore shifted toward engineering model microorganisms, such as *Escherichia coli*, *S. cerevisiae*, *C. glutamicum*, and *Bacillus subtilis*, which have well-understood genetic backgrounds, abundant biological parts, and devices, and for which comprehensive genetic modification tools exist [4,34] (Table 3).

**Table 3.** Characteristics of representative model and nonmodel microbial chassis cells

Class	Strain	Growth	Safety	Genome (Mb)	Substrates	Genome manipulation tools	Products	Reference
Model microbes	*E. coli*	Facultative aerobic	Not GRAS	4.64	Glycerol, pentose, hexose, starch	Various tools	Alcohols, fatty acids, terpenoids	[68,124,169]
	*S. cerevisiae*	Facultative aerobic	GRAS	11.8, 16 chromosomes	Starch, sucrose, hexose	Various tools	Terpenoids, natural, products	[40,170]
	*C. glutamicum*	Facultative aerobic	GRAS	3.28	Sugars, alcohols, organic acids	HR, CRISPR-Cas9, CRISPR-Cpf1/dCpf1	Alcohols, aminol acids, hyaluronan	[171,172]
	*B. subtilis*	Facultative aerobic	GRAS	4.2	Glucose, acetate, citrate	CRISPR-Cas9, CRISPR-dCas9-α/ω, CRISPR-Cpf1/dCpf1	Enzymes, isobutanol, 2,3-BDO	[173–175]
Nonmodel microbes	*Y. lipolytica*	Facultative aerobic	GRAS	20.5, 6 chromosomes	Glucose, glycerol, sucrose, starch, inulin, cellobiose	NHEJ, ZFNs, TALENs, CRISPR-Cas9, (CRISPRi/CRISPRa)	Lipid, FAAEs, terpenoids, alkanes	[98,176]
	*Rhodotorula toruloides*	Facultative aerobic	GRAS	20.2	Glucose, xylose, cellobiose	CRISPR-Cas9, RNA interference	Lipid, biodiesel, carotenoids, enzyme	[162,177–179]
	*Scheffersomyces stipitis*	Facultative aerobic	GRAS	15.4, 8 chromosomes	Glucose, fructose, xylose, mannose, galactose, cellobiose	CRISPR-Cas9	Shikimic acid, ethanol, vitamin B_9_	[180,181]
	*Pichia pastoris*	Facultative aerobic	GRAS	9.6, 4 chromosomes	Glucose, glycerol, l-rhamnose, methanol	HR, CRISPR-Cas9	Enzyme, terpenoids, alcohols	[48,49,182]
	*Z. mobilis*	Facultative anaerobic	GRAS	2.2, 4 plasmids	Glucose, sucrose, fructose	HR, CRISPR-Cas9, CRISPR-Cas12a, endogenous type I-F CRISPR-Cas system	Ethanol, isobutanol, 2,3-BDO, PHB	[83,84,87,88]
	*C. autoethanogenum*	Obligate anaerobic	Not GRAS	4.4	CO, CO_2_	Endogenous type I-B CRISPR cluster	Acetate, ethanol, 2,3-BDO; PHB	[183]
	*C. acetobutylicum*	Obligate anaerobic	Not GRAS	4.1	Glucose	CRISPR-Cas9/dCas9	Acetone, ethanol, butanol	[184,185]
	*H. bluephagenesis*	Aerobic	Not GRAS	4.09	Glucose, butyrate	CRISPR-Cas9 CRISPRi CRISPR/AID	PHA, ectoine, l-threonine	[102,186]
	*V. natriegens*	Facultative anaerobic	GRAS	Chr: 3.24, Chr: 1.92	Glucose, fructose, glycerol, citrate	CRISPR-Cas9 CRISPRi Cre-loxP system	PHA, alanine carotene melanin	[187]

The model bacterial species *E. coli* is often used as a chassis to develop MCFs for the production of bulk chemicals such as l-alanine [35], 1,3-PDO [36], d-lactic acid [37], succinic acid [38], and 1,4-butanediol [39] because of its rapid growth and simple nutritional requirements. Owing to the lack of isoprene synthesis pathways required for the synthesis of terpene compounds, it is often used for the synthesis of simple terpene natural products through the 2-C-methyl-d-erythritol 4-phosphate (MEP) pathway. *S. cerevisiae* has a natural mevalonate (MVA) pathway, which leads to greater synthesis of precursor substances than does the MEP pathway [40]. Therefore, *S. cerevisiae* is commonly used for the synthesis of natural products [40]. *C. glutamicum*, a model microorganism for amino acid production, has also been engineered to produce natural products and nonessential amino acids. Owing to its strong secretion ability, *B. subtilis* is used to produce natural products including monosaccharide derivatives such as scyllo-inositol, terpenoids such as squalene, and active polysaccharides such as hyaluronic acid and chondroitin sulfate [41–44].

Therefore, these model microorganisms modified by metabolic engineering could be referred to as third-generation industrial microbial cell factories (MCF 3.0). This class not only possesses a series of technology platforms, including gene design, synthesis, editing, rich biological parts and devices, and massive databases but also establishes relatively mature commercial production infrastructures and business models, making them important chassis cells in biomanufacturing. However, it is still challenging to rapidly transform these model microorganisms into the desired MCFs for biomanufacturing.

### Direction and strategies of metabolic engineering

**Fig. 3.**
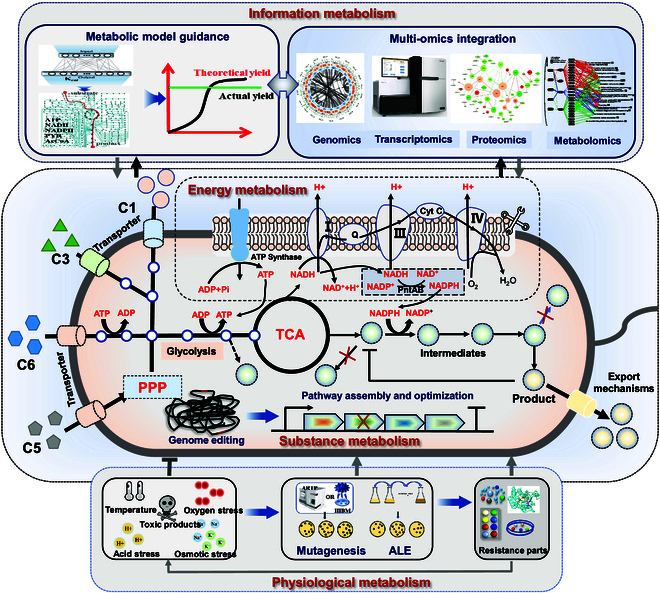
The direction and strategy of material, energy, physiology, and information metabolism optimization in MCFs.

Substance and energy metabolism are current primary modification targets for strain construction and improvement (Fig. 3). Substance metabolism modification can improve the utilization rate of substrates in cell factories, broaden their substrate utilization diversity, reduce the generation of redundant byproducts, and maximize the flow of carbon metabolism toward the target product. Energy metabolism modification can provide sufficient energy and cofactors for the chemical production process of cells, balancing the reducing power and promoting efficient production of target compounds.

### Broadening substrate utilization

To actively respond to the impact of fluctuating raw material prices, developing microbial chassis cells with different substrate utilization capabilities is necessary. Lignocellulose is the most abundant renewable resource on Earth and the main raw feedstock for second-generation biorefining [45]. After pretreatment and enzymatic hydrolysis, the monosaccharides are released for utilization and fermentation by microorganisms. These monosaccharides include C5 sugars such as xylose and arabinose, as well as C6 sugars such as glucose, mannose, and galactose. However, the majority of microorganisms can use only specific sugars as carbon sources. Therefore, it is crucial to achieve economic biochemical production by coutilizing multiple carbon sources of lignocellulosic hydrolysates by introducing metabolic pathways for different carbon sources to expand the substrate utilization spectrum. Combining adaptive evolution after the introduction of substrate utilization pathways and transport systems, efficient substrate utilization cell factories have been obtained (Fig. 3). For example, the production of ethanol from xylose has been achieved by introducing exogenous xylose reductase and xylitol dehydrogenase or heterologous xylose isomerase pathways into yeast [46].

In addition to the use of lignocellulose as a raw material, C1 molecules such as CO_2_, methanol, and formate are also highly promising feedstocks [47]. At present, microorganisms that can utilize C1 molecules include those that use methyl compounds as the sole carbon source for the growth and metabolism of natural methyl nutrient microorganisms, as well as methanol-consuming strains such as *E. coli* and *C. glutamicum* obtained through metabolic engineering and adaptation. Through metabolic engineering of natural yeast, the synthesis of primary metabolites such as ethanol, isobutanol, and 2,3-butanediol (2,3-BDO), as well as secondary metabolites such as polyketones, terpenes, and fatty acid derivatives, has been achieved [48–52]. Carbon dioxide is the main raw material for third-generation biorefineries. Designing and developing microbial chassis cells to directly use CO_2_ to produce biofuels and biochemicals can greatly alleviate energy depletion and environmental problems [53]. The naturally occurring carbon sequestration pathway has been explored and applied to various model microorganisms, and autotrophic growth of *E. coli* has been achieved through continuous laboratory evolution by introducing RuBisCO carbon sequestration enzymes [54]. In addition, various artificial carbon sequestration pathways have been developed and applied [55].

### Balancing cofactors and energy

Cofactors and adenosine triphosphate (ATP) are key regulatory factors in energy metabolism and are also important for maintaining cell growth and homeostasis [56]. Cells produce reducing power and ATP when metabolizing substrates such as glucose, and require them when synthesizing biochemicals. If the cofactor and ATP supply is imbalanced, the conversion rate and synthesis efficiency of the products will be affected.

Intracellular ATP is regulated mainly by ATP-related enzyme metabolism and oxidative phosphorylation levels [57]. The activities of intracellular NADH [reduced form of nicotinamide adenine dinucleotide (oxidized form)], the electron transfer chain, and ATP synthase are the main sites for regulating intracellular ATP levels. To increase the ATP supply, metabolic engineering can be used by adding ATP energy substrates, controlling the generation and consumption of pH and ATP, and regulating the electron transfer chain. After the energy substrate citric acid was added to the fermentation system, the titer of poly-γ-glutamic acid (PGA) in *B. licheniformis* increased to 35 g/l [58].

The following 4 strategies can be implemented to optimize the supply of reducing power in the production process: regulating the synthesis and regeneration of the intracellular reducing power, changing the cofactor preference of key enzymes, consuming redundant cofactors, and introducing nonnatural cofactors. In regulating the synthesis and regeneration of intracellular cofactors, activating the combined expression of transhydrogenase and NAD (nicotinamide adenine dinucleotide) kinase can effectively increase intracellular NADPH (reduced form of nicotinamide adenine dinucleotide phosphate) levels and subsequently isobutanol production [59].

In addition, the Entner–Doudoroff (ED) pathway is often used as an alternative pathway to Embden–Meyerhof–Parnas (EMP) because of its efficient NADPH generation. For example, the ED metabolic pathway derived from *Z. mobilis* was introduced into *E. coli,* and the expression of related genes was regulated via ribosome binding site (RBS) libraries. The NADPH level in the recombinant *E. coli* was 25 times greater than that in the wild type, effectively increasing the production of terpenoids [60]. This strategy was also suitable for increasing isobutanol production in *E. coli* and *C. glutamicum* [61].

### Improving robustness

MCFs are subjected to various physiological or physiochemical stress factors during fermentation using the inexpensive renewable resources, including changes in temperature, pH, oxygen, and osmotic pressure as well as changes in the concentrations of substrates, inhibitors, and toxic intermediates or byproducts. These stress factors can slow microbial metabolism and cell growth and sometimes even lead to the complete loss of production performance (Fig. 3).

Traditional mutagenesis and adaptive laboratory evolution (ALE) are effective strategies for improving strain robustness [62]. This method is widely used in both model and nonmodel chassis cells to improve the tolerance to toxic substances during the production process. The most common method is to increase the tolerance of chassis cells to toxic products to increase yield. For example, a serine-sensitive production strain lacking the l-serine degradation pathway was subjected to ALE to improve l-serine tolerance with gradually increasing concentrations from 3 to 100 g/l, and evolved strains with excellent growth performance at a titer of 50 g/l l-serine were isolated. These mutant strains presented improved serine production [63]. ALE can also be combined with genome sequencing and transcriptome analysis to further understand tolerance mechanisms at the genetic level [64,65]. In addition, ALE can effectively improve the metabolic disorders of microbial chassis cells. Through adaptive evolution of lipolytic yeast, ALE can effectively improve the metabolic disorders caused by the reductive tricarboxylic acid (TCA) cycle, thereby increasing cell growth ability and succinic acid production [66]. Therefore, ALE plays a crucial role in the development of industrial chassis cells.

Owing to the complicated screening process, prolonged cycles, and tedious workload involved in ALE, the pursuit of stress-resistant biological parts for rational design and engineering of microbial cells to increase their robustness has emerged as an alternative strategy to increase stress resistance (Fig. 3). With the accumulation of omics datasets, an increasing number of stress-resistant elements are being identified and applied for the construction of robust microorganisms. These stress-resistant elements mainly include genes related to cell walls and membranes, DNA repair, oxidative stress, compatible solutes, energy production and signal transduction, as well as efflux pumps, heat shock proteins, and global transcription factors [67], which can be used for genetic engineering to rationally modify strains for improved robustness.

For example, the tolerance of *E. coli* to organic alcohols and product yield were effectively improved by enhancing cell membrane biosynthesis and modifying membrane transport proteins [68–70]. In addition, heat shock proteins (GroELS, DnaK, DnaJ, HtpJ), DNA repair-related genes (RecA, UvrD), oxidative stress genes (Fpr, Gsh1, Glt1), and global transcription factors (CRP, RpoD) can also increase microbial tolerance to inhibitors [71–74]. Sulfur-containing amino acids are important essential amino acids, and their concentrations are closely related to microbial stress resistance. Hydrogen sulfide, an important intermediate product of sulfur metabolism, also plays an important role as an endogenous signaling molecule in response to reactive oxygen species (ROS) stress [75].

### Rewiring the metabolic network

Owing to the varying number of genes involved in the biosynthetic pathway, the continual development of chassis strains that are proficient in producing high-yielding intermediate products reduces the complexity of pathway reconstruction greatly. This ensures an adequate supply of precursors, enabling the adoption of tailored strategies to optimize chassis cells, regulate metabolic balance, and ultimately facilitate the efficient construction of MCFs (Fig. 3). Genome-wide metabolic models were used to search for genetic targets for metabolic engineering, efficiently design a metabolic pathway reconstruction library, and combine with high-throughput biosensors to train different machine learning algorithms. A data generation cycle promoted the synthesis ability of aromatic amino acids in engineered yeast, resulting in 74% and 43% increases in tryptophan production titer and yield, respectively [76].

When reshaping the metabolic network of chassis cells, attention should be given to the interactions among various metabolic pathways to reduce the disturbance of common substances such as coenzymes and the energy supply caused by the introduction of biosynthesis pathways. Additionally, the introduction of heterologous pathways may have a negative influence on the physiological status and growth rate of chassis cells, as these heterologous products or intermediates may have certain toxicity toward the chassis cells.

Bioinformatics strategies are evolving with the continuous increase in high-throughput data such as whole-genome sequences of microorganisms, enabling comprehensive and systematic analysis, design, and regulation of microbial physiological metabolic functions. The emergence of the genome-scale metabolic network model (GSMM) based on genome sequence annotation and detailed biochemical information integration can help accurately predict and understand the mechanisms of microbial intracellular metabolism. At present, strain design and phenotype prediction analyses based on genome-scale metabolic models have been performed in various microorganisms, such as *E. coli*, *B. subtilis*, *C. glutamicum*, and *S. cerevisiae*. After years of revision and improvement, the accuracy of model predictions has improved. Through the combination of genome-wide metabolic modeling, genetic targets for strain modification can be predicted, and recombinant strains with improved titers, yields, and rates of the targeted products can be generated [77–79]. For example, the ability of engineering yeast to produce aromatic amino acids has been improved by combining high-throughput biosensors with the guidance of GSMM, leading to an increase in tryptophan production and yield of 74% and 43%, respectively [76].

## Synthetic Biology-Guided Industrial Microbial Chassis Cell Modification

However, model microorganisms usually have innate disadvantages when using low-cost feedstocks, such as poor fermentation performance due to their low tolerance to toxic lignocellulosic hydrolysate, which limits the industrial application of model species in lignocellulosic biochemical production. With the development of systems and synthetic biology, more nonmodel microbial chassis cells with excellent industrial characteristics have been explored, such as *Z. mobilis*, *Halomonas bluephagenesis, B. licheniformis, Clostridium autoethanogenum, C. acetobutylicum, Vibrio natriegens,* and unconventional yeasts (Table 3), which can be used as chasses for economic biochemical production.

Synthetic biology provides powerful tools for designing, synthesizing, and reconstructing biological parts, circuits, and modules into functional synthetic cell factories. The breakthroughs in effective gene editing, synthesis, and assembly tools can facilitate the construction of robust and efficient industrial strains, and MCFs can be further improved to meet industrial needs.

Bioethanol is currently one of the most widely used bioenergy sources in the world. Its large-scale industrial production is achieved mainly by *S. cerevisiae* using grain crops such as corn or economic crops such as sugarcane. However, producing bioethanol from crops faces the challenges of competing with humans for food usage, which is not conducive to sustainable development. Therefore, the use of nonfood feedstocks for bioethanol production is a future trend. *Z. mobilis* is a nonmodel polyploid ethanologenic gram-negative bacterium with many industrial merits and unique physiological properties [80]; it is the only known microorganism that can utilize the ED pathway under anaerobic conditions [81] and has excellent characteristics compared with *S. cerevisiae*, such as a high sugar uptake rate, high ethanol yield, and ethanol tolerance [82]. DuPont has achieved the industrial production of 30 million gallons of cellulosic ethanol per year via C5-utilizing recombinant *Z. mobilis* [45]. In addition, efficient genome-editing tools based on heterologous clustered regularly interspaced short palindromic repeats (CRISPR)–CRISPR-associated protein 12a (Cas12a), endogenous type I-F CRISPR-Cas and associated repair pathways such as microhomology-mediated end joining (MMEJ), as well as a genome-wide iterative continuous editing (GW-ICE) system have been developed [83–86]. Many MCFs have been constructed for the production of acetaldehyde, lactate, l-alanine, l-serine, acetoin, 2,3-BDO, isobutanol, l-malate, succinic acid, poly-3-hydroxybutyrate (PHB), farnesene, and ethylene [21,87–97].

Succinic acid is a promising platform chemical used for the production of biomaterials such as 1,4-butanediol, γ-butyrolactone, tetrahydrofuran, and polybutylene succinate (PBS), which is also listed by the US Department of Energy as one of the 12 top platform chemicals. *E. coli,* in addition to some natural succinic acid-producing strains, is generally the most commonly used chassis cell for succinic acid production. Although these natural bacteria can achieve high yields and titers of succinic acid production, the fermentation process normally requires obligate anaerobic conditions with the addition of alkali to maintain neutral pH conditions, which increases costs in industrial production. *Y. lipolytica* has a strong ability to synthesize acetyl-CoA (coenzyme A), providing sufficient precursors for succinic acid production. High-efficiency succinic acid at low pH was achieved at a titer of 111.9 g/l by reconfiguring the reductive TCA cycle in *Y. lipolytica* [66]. Many important synthetic biology tools and components have been developed and applied in *Y. lipolytica*, such as DNA assembly technology, genome editing technology, and computational tools [98]. The development of these tools has further accelerated the construction of MCFs using *Y. lipolytica* as the chassis cell [98].

PHAs are polyesters synthesized by a wide range of bacteria and have been developed into various environmentally friendly plastic products [99]. *H. bluephagenesis* TD01 is an extreme microorganism that can accumulate ~80% (wt%) poly-β-hydroxybutyrate (PHB) naturally. Additionally, *H. bluephagenesis* has been engineered to produce 3-hydroxybutyrate, 3-hydroxypropionate, and 4-hydroxybutyrate [99–101] and other biochemicals, such as lysine, ectoine, cadaverine, and 5-aminolevulinic acid. Extremophiles such as halophilic bacteria provide a model and paradigm for the development of next-generation industrial biotechnology (NGIB) [102]. Lactic acid, another monomer of bioplastics, is not limited to natural microbial synthesis. It has been synthesized through synthetic biological methods in both model and nonmodel microorganisms [90].

Functional sugars are a type of carbohydrate that cannot be digested and absorbed by the human gastrointestinal tract and have special properties, such as sweetness flavor or the ability to promote probiotic growth. They mainly include functional oligosaccharides, sugar alcohols, and starch. These sugars can be used as functional food additives or raw materials alternatives to sucrose to meet the needs of special populations, such as those with diabetes and obesity. At present, its biosynthetic technology mainly includes enzymatic catalytic conversion technology and microbial fermentation technology. Food-grade enzyme expression systems were developed using *B. subtilis* and *C. glutamicum* as chassis strains, and various monosaccharides, dioxygenases, and branched-chain ketose compounds were generated by exploring different enzyme elements [103–105]. *E. coli*, *S. cerevisiae*, *C. glutamicum*, and *B. subtilis* were all designed and developed as chassis cells for human milk oligosaccharide (HMO) synthesis.

Artificial MCF synthesis of artemisinin is a landmark achievement of synthetic cell factories for biomedicine. Owing to their potential applications and great challenges, the construction of MCFs for natural products has attracted considerable attention. The high yield of terpenoid compounds in the cell factory when *Y. lipolytica* was used as the substrate increased the titer of squalene by a factor of 1,300 [106]. The biosynthesis of cannabidiol was achieved via the use of *S. cerevisiae* as the chassis cell [107]. The reconstruction and analysis of the biosynthesis pathway of Taxol have laid a solid foundation for elucidating its complete pathway [108,109].

The achievements in both nonmodel and model microorganisms led to the formation of fourth-generation industrial microbial cell factories (MCF 4.0), which can play important roles in bioenergy, biomaterials, bioplastics, biomedicines, food, and nutrients. Disruptive enabling technologies are the key to reprogramming biological genes, circuits, pathways, networks, and other components to continuously iterate and upgrade MCFs to accelerate the construction and application of efficient cell factories.

### Enriching biological components and developing enabling technologies

**Fig. 4.**
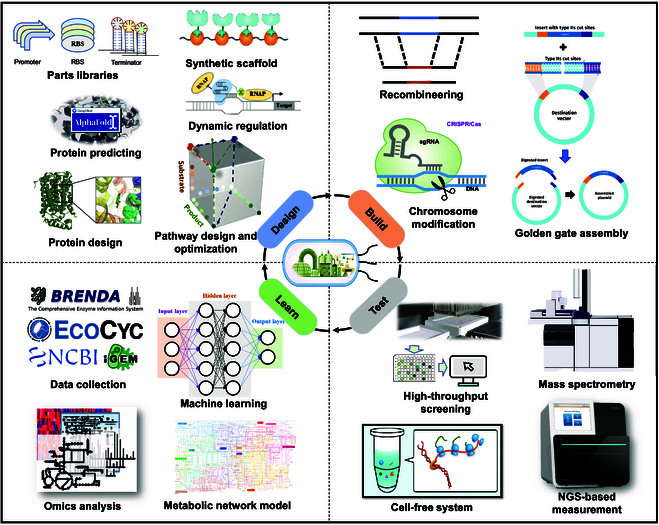
Enabling technologies and applications of synthetic biology for the construction and optimization of MCF 4.0.

Owing to the high complexity of cells, artificially implanted biological elements, circuits, or systems are influenced by the existing metabolic and regulatory pathways within the cells. Therefore, the continuous expansion of biological components and improvements in enabling technologies are needed. These include the excavation and identification of biological parts, circuits, and modules; the understanding of cell metabolism and regulatory networks among different chassis cells; the development of design theories and tools; and the improvement of high-throughput automatic assembly and testing methods for accelerating the development of microbial chassis cells and the construction of efficient MCFs (Fig. 4). Finally, enabling biotechnology tools need to be integrated with automation and AI for further applications.

### Characterization and applications of biological parts

Biological parts are one of the fundamental elements in synthetic biology and constitute the cornerstone of synthetic biology. Standard biological parts include DNA sequences such as promoters, terminators, transcription units, plasmids, conjugation transfer elements, transposons, and protein coding regions, as well as RNA sequences such as RBSs and protein domains. The excavation, identification, and modification of biological components is an important research direction in the field of synthetic biology.

An increasing number of biological parts, including promoters and RBSs, have been applied to regulate metabolic network flux, increase target product yield, and reduce byproduct generation. It has become an inevitable trend to build a promoter library with a range of strengths, as is the case for yeast and *E. coli* [110]. In addition, artificial RBS and untranslated region sequences with different translation levels can be predicted and designed using computational tools [111].

For the convenience of different levels of design, researchers have developed corresponding analysis tools for different nonstandard biological components, such as Softberry software and online analysis websites for promoter prediction analysis and the Database for Prokaryotic OpeRons (DOOR) for prokaryotic operator prediction analysis [112,113]. In addition, a series of biological element algorithms (Algorithm) tools, metabolic pathway constructions, and protein expression processes (Workflows) have been developed. These include the RBS Library Calculator for predicting translation rates and controlling expression levels; the RBS Library Calculator for predicting expression changes, optimizing expression levels, and designing libraries; the Operator Calculator for predicting the expression of operons and multiple proteins; the ELSA Calculator for regulating multiple genes; the Syth Success Calculator for optimizing DNA synthesis; the NRP Calculator for mining and designing nonrepetitive elements; and the Riboswitch Calculator for predicting riboswitches and designing RNA sensors [114,115].

### Gene circuits

With an increasing number of bioparts being excavated and characterized, redesigning and constructing bioparts enables artificially designed biomolecular pathways to regulate metabolic networks in MCFs, which has enormous potential in biological manufacturing. Gene circuits comprise 2 basic types: transcriptional gene regulatory circuits and protein-based signaling circuits.

Transcriptional gene regulatory circuits control gene expression through transcription. The widely used transcriptional gene regulatory circuits are T7 RNAP, LacI, and TetR. The T7 expression system established on the basis of the specific and efficient recognition between T7 RNAP and the P_T7_ promoter is currently one of the most powerful expression systems for exogenous proteins in *E. coli* and has been widely used for protein expression [116,117]. The TetR-derived promoter has also been used for the production of biochemicals, such as isobutanol and acetoin [88,93]. Promoter–transcription factor circuits have also been used for the dynamic regulation of MCFs. Transcription factors can bind to specific metabolites and promoter region DNA, thereby activating or inhibiting downstream gene expression. Acyl-CoA responsive dynamic sensor regulatory system (DSRS) was developed to regulate the expression of fatty acid ethyl ester production genes, resulting in a 3-fold increase in fatty acid ethyl ester production to 1.5 g/l [118].

With in-depth research on the quorum-sensing (QS) mechanism of microorganisms, dynamic regulation based on QS has also been used to decouple cell growth and production processes. By regulating the expression levels of the cell density-dependent genes *luxI* and *luxR* and changing the switching time of trigger gene expression, this system has been applied to the production of 1,4-butanediol and bisabolene and the redirection of the TCA cycle to isopropanol through trigger switching [119–121].

### Developing and optimizing genome-editing strategies

For industrial applications of MCFs, the chromosomal expression of biosynthetic genes is often favored over plasmid-based expression because of the instability of plasmids. Zinc-finger nucleases (ZFNs) and transcription activator-like effector nucleases (TALENs) constitute a powerful class of tools for genetic modifications in the past 10 years, and with the elucidation of the mechanisms of the CRISPR-Cas system, various CRISPR-based technologies have been developed as powerful genome editing tools for MCF construction [122].

The CRISPR-Cas system originates from the immune system of microorganisms and has recently attracted great interest as a gene editing tool because of its precision and rapid characteristics compared with those of conventional tools. For model microorganism applications, CRISPR-Cas9 was first introduced into *E. coli* for gene editing [123] and then optimized to achieve simultaneous editing of 3 genes [124]. To increase the curing and editing efficiency of plasmid editing, a Rock-Paper-Scissors strategy, a robust and fast iterative genome-editing strategy, was designed and applied in *E. coli* and *K. pneumoniae* [125]. Additionally, a mature CRISPR-Cas editing system was constructed and applied in other model microorganisms (Table 3).

As important industrial chassis cells for cellulosic ethanol production, gene editing systems based on exogenous CRISPR-Cas12a and endogenous I-F-type CRISPR-Cas systems have been successfully developed in *Z. mobilis*, as well as CRISPRi technology based on dCas9 and the guide RNA (gRNA) design network tool CRISpy-pop [83,84,126], providing diverse genetic tools for genome editing and modification and for successfully constructing efficient cell factories for d-lactic acid and acetoin production [90,93]. Transposon mutation, homologous recombination, the CRISPR-Cas9 editing system, and a multigene editing system have been established in *V. natriegens* [127,128], which has accelerated the process of metabolic engineering and genome simplification optimization and achieved the production of recombinant proteins, melanin, carotenoids, and violets [129–134]. In addition, genome-editing systems have been established in some nonmodel microorganisms, and the synthesis of biochemicals has been achieved, accelerating the industrial application of nonmodel microbial chassis cells [135,136].

### Genome minimization

Genome minimization is an important strategy for the rational construction of excellent chassis cells. Cell energy consumption is reduced by knocking out redundant genes, allowing more energy to be used for target product production [137]. Various microorganisms have demonstrated excellent characteristics through genome minimization, such as increased biomass, genetic stability, transformation efficiency, and product yield [137]. For example, 663 kb of genomic DNA was removed from *E. coli* MDS42, resulting in a 2-order increase in transformation efficiency compared with that of the parental strain [138]. After 1,670 kb of genomic DNA was deleted, the *E. coli* DGF-298 genome was more stable, with a higher growth rate and cell density. From the perspective of biological product synthesis, the simplified *B. subtilis* BSK814G2 genome lacks 814.4 kb of genomic DNA, but the cells accumulate 115.2 mg/l guanosine (~4.4 times greater than that of the original strain). The minimization of *B. subtilis* BSK756T3 resulted in a 5.2-fold increase in the concentration of intracellular 151.2 mg/l thymidine [139]. In addition, after 7.7% of the genomic DNA in *Pseudomonas mendocina* NK-01 was deleted, the production of PHA and alginate oligosaccharides increased by 114.8% and 27.8%, respectively [139]. Therefore, through genome minimization, a microbial chassis with excellent traits and high yield can be obtained.

### High-throughput screening

High-throughput screening can be used to obtain desired mutants from large variant libraries quickly and efficiently. With the development of molecular biology and gene editing technology, genetic engineering methods continue to be iterated and innovated, resulting in different mutant libraries. Combining traditional microplate-based screening methods with automated equipment can reduce labor intensity and improve screening efficiency to 10^3^ to 10^5^ colonies per day, and has been successfully used in various industrial microbial strain screening methods [140,141].

In addition, fluorescent markers, mass spectrometry, and single-cell Raman spectroscopy (SCRS) have also been introduced on the basis of their unique characteristics. SCRS technology can recognize panoramic information at the level of living single cells and nonlabeled states, thereby distinguishing complex functional phenotypes. It has advantages such as being fast and economic, can be coupled with downstream omics research, and is considered a new single-cell phenotype recognition technology [142]. Flow cytometry screening, such as fluorescence-activated cell sorting (FACS), can analyze and sort individual cells on the basis of specific criteria. However, the application of FACS is limited to analyzing fluorescence signals related to intracellular target products or metabolites bound to membranes and cannot be used for identifying cells that overproduce secreted metabolites and secreted extracellular enzymes. Mass spectrometry is highly sensitive and does not require specific labeling, which can provide key technical support for high-throughput screening of MCFs without epigenetic traits [143].

Compared with the above screening methods, droplet microfluidics, which mainly involve the formation of monodisperse microdroplets through the shearing of 2 incompatible fluids, can monitor quantitative single-cell metabolite concentrations with increased screening efficiency. More precise operations such as droplet incubation, fusion, division, detection, and sorting in the chip can be achieved. To better achieve high-throughput and automated continuous cultivation of microbial chassis cells to obtain mutant strains with excellent characteristics, a microbial microdroplet culture system (MMC) was developed by combining microfluidic technology and photoelectric sensing and automation technology for parallel cultivation and growth curve monitoring of microbial droplets. This accelerated the evolution of numerous microbial chassis cells, greatly improving the efficiency of obtaining excellent phenotype samples, such as substrate utilization ability, solvent tolerance, and robustness of microbial substrate cells [144,145].

### Artificial intelligence

Computer-aided design (CAD) of biomolecules with specific functions greatly improves the ability and quality of biological parts and systems. The rational design of biological parts, circuits, pathways, and systems can be achieved through AI or computational biology to achieve better biological expression and higher production efficiency. For example, AI algorithms can be used to design and optimize promoter sequences, thereby increasing the expression level of target genes and reducing side effects. A machine learning model for predicting promoter strength in *E. coli* was designed on the basis of artificial neural networks and support vector machines [146].

Moreover, computational biology methods can also be used to simulate and predict the expression levels and functions of enzymes in different environments. Design methods and tools such as FireProt, GRAPE-WEB, PROSS, Funclib, ABACUS2, the Swiss Model, ConSurf, and ROSIE have been developed based on the selective modification and stability enhancement of enzymes, greatly improving the efficiency of enzyme design [147–153]. With the application of AI, an increasing number of key enzymes and metabolic pathways in metabolic networks have been identified by analyzing large amounts of metabolic datasets. Computational biology methods can also be used to simulate and predict the performance of metabolic pathways under different conditions, thereby helping to optimize the design of metabolic engineering and better guiding the construction and development of efficient MCFs.

## Creation of Customized Artificial Cell Factories

The development of a knowledge base of microbial cells and the technological advancements in AI and automation have made rational design, synthesis, and testing of chassis cells more intelligent, greatly reducing the trial-and-error frequency of industrial chassis cells during their development process. In addition, the rapid accumulation of omics data and AI has led to the efficient discovery and analysis of many genes of unknown function in the genome. Customized industrial chassis cells can be obtained by modifying genotype-associated phenotypes, which can also be referred to as fifth-generation industrial microbial cell factories (MCF 5.0).

The development of sequencing technology and the rapid growth of biological data resource models have brought new understanding and development opportunities to the design of MCFs. The customized design of MCFs can be based on massive amounts of data resources and adopt biological design automation. The automated design of cell factories can be completed through a series of algorithms to predict and screen biosynthetic pathways, the design of regulatory components and pathways, and the adaptability of pathways and the chassis. On the basis of data- and knowledge-driven approaches, intelligent design and automated high-throughput construction testing are assisted to further develop the ability to synthesize biobased products via the use of diverse chassis cells, such as those of microorganisms, animals, and plants. This enables comprehensive development from the local modification of genetic components to new creations, spanning from the local optimization of genetic networks to overall adaptation, local genome replacement to de novo design and synthesis, and the passive observation of cellular functions for precise control [154]. The efficient and intelligent creation of enzymes and cell factories promotes the green and sustainable development of the bioeconomy, especially cell factories, which are regarded as “chips” that are directly related to research and industrial development in biological manufacturing.

## Future Perspectives of Industrial Digital MCFs

 Achievements have been made in constructing cell factories to synthesize biochemicals using model or nonmodel microbial chassis cells by combining systems biology, synthetic biology, and metabolic engineering technologies. However, the majority of target product syntheses have not yet met the requirements of industrial production at present, and there is an urgent need to achieve breakthroughs in yield and productivity. Therefore, it is necessary to further explore and study the key biological components in the biosynthetic pathway of biochemicals, design and reconstruct the metabolic mode of chassis cells, optimize the adaptability of synthesis pathways and metabolic networks, and combine process engineering technology with intelligent control strategies to support fermentation processes. In the era of systems and synthetic biology, the constantly evolving biotechnology and rapidly developing IT will provide abundant tools and resources for the construction and optimization of efficient MCFs in biological manufacturing.

The construction of an efficient cell factory integrates disciplines such as stoichiometry, computer simulation, and biotechnology, especially with the current development of synthetic biotechnology, making it possible to transform living organisms to serve humanity. In the design and assembly process of cell factories, it is necessary not only to evaluate the feasibility of metabolic pathways through stoichiometry but also to optimize and design more efficient synthesis pathways and components through computer simulation and other tools. On this basis, biotechnology is used to assemble and fine tune the synthesis module, ensuring a sufficient supply of precursors in the metabolic pathway, smooth metabolic flow, cofactor balance, and feedback inhibition release, thereby obtaining an efficient cell factory for synthesizing target chemicals. With the advancement of synthetic biotechnology, many expression elements and regulatory methods have been developed, and these tools will gradually be applied to the construction and optimization of artificial synthetic cell factories.

AI is playing an increasingly important role in synthetic biology. In the past few years, AI has achieved great success in protein design. With their powerful feature extraction, data statistics, and function fitting capabilities, advanced AI models learn basic features and interaction relationships from existing protein structure and sequence data and fit generalized function models for application in various protein design tasks. Taking AlphaFold as an example, combining advanced machine learning techniques and biophysical knowledge enables AlphaFold to improve the accuracy of protein structure prediction, thereby accelerating the progress of biological science [155]. The predictive accuracy of the powerful protein–protein interactions of AlphaFold3 also represents a major step forward in predicting the structure of biomolecules [156]. The prediction and development of proteins by AI can be further applied to the development and optimization of efficient MCFs, promoting the development of cell factories.

With the further development and cross-integration of automation, IT, and biotechnology, various technologies are expected to become increasingly mature. The understanding of chassis cells is gradually increasing, the library of biological components is constantly expanding, and the design, construction, optimization, and adaptation of artificial metabolic pathways are becoming more convenient and practical. Fermentation process optimization and control are becoming increasingly intelligent, which will lead to breakthroughs in the efficient, green, and low-cost synthesis of customized artificial cell factories for economic biochemical production and commercialization.

## Conclusion

The discovery and creation of cell factories is a long process, from initially requiring a large number of human resources to accelerating the iteration and updating of MCFs with the emergence of efficient and effective tools. The development of MCFs is undergoing diverse processes, and the application and development of model and nonmodel microorganisms have promoted the industrial application of MCFs. The emergence of synthetic biology is a revolutionary subversion of MCFs. However, in the era of AI, the construction and optimization of MCFs not only require advanced biotechnology methods but also rely on digital technologies with stronger simulation and computing capabilities to reduce time and material costs. With the support of multiple biotechnologies and AI, MCF 5.0 will demonstrate better performance and a more intelligent form.
